# Co-existence of Ménétrier’s Disease and Collagenous Colitis: A Diagnostic and Therapeutic Challenge

**DOI:** 10.1007/s11606-026-10301-0

**Published:** 2026-03-12

**Authors:** Shigeto Mashiko, Daiki Murata, Ryohei Takahashi, Daisuke Fukushi, Takahiro Ohara, Katsutoshi Furukawa

**Affiliations:** 1 Division of Geriatric and Community Medicine, Faculty of Medicine, Tohoku Medical and Pharmaceutical University, Sendai, Miyagi Japan; 2 Division of Gastroenterology, Faculty of Medicine, Tohoku Medical and Pharmaceutical University Hospital, Sendai, Japan

**Keywords:** Ménétrier’s disease, collagenous colitis, hypertrophic gastropathy, persistent nausea, protein-losing gastroenteropathy

## INTRODUCTION

Ménétrier’s disease is a rare hypertrophic gastropathy characterized by enlarged gastric folds, foveolar hyperplasia, and protein-losing gastroenteropathy. Its insidious onset and non-specific symptoms, such as nausea and vomiting, can delay the diagnosis in the absence of overt protein loss. In this case, overlapping symptoms of two independent gastrointestinal disorders, Ménétrier’s disease and collagenous colitis, obscured the evolving clinical picture until hypoproteinemia prompted renewed diagnostic attention and the diagnosis of Ménétrier’s disease. The favorable response of this case to a histamine-2 receptor antagonist (H2RA) for Ménétrier’s disease raised questions regarding disease mechanisms and potential therapeutic implications. This report is the first to describe co-existing Ménétrier’s disease and collagenous colitis.

## CASE PRESENTATION

A 60-year-old man presented with a 1-month history of worsening nausea, vomiting, and diarrhea, as well as 5 kg of unintentional weight loss and bilateral leg edema. He had been using vonoprazan (potassium-competitive acid blocker (P-CAB)) with mosapride citrate (5-HT4 receptor agonist) and acotiamide (gastroprokinetic agent through acetylcholinesterase inhibition and muscarinic receptor antagonism not available in the USA) for gastroesophageal reflux disease and functional dyspepsia. For his 30-year history of type 2 diabetes mellitus he was taking metformin, dapagliflozin, mitiglinide (short-acting insulin secretagogue), glimepiride, and a combination of insulin glargine and lixisenatide (glucagon-like peptide-1 receptor agonist [GLP-1RA]). He denied significant alcohol use and had a 40-pack-year smoking history; he recently quit smoking because it exacerbated his nausea. Family history was significant for diabetes and pancreatic cancer in his father.

One year before presentation, contrast-enhanced abdominal computed tomography (CT) was performed to investigate possible pancreatic cancer because of his family history, smoking history, worsening glycemic control, and persistent epigastric discomfort. It incidentally revealed gastric wall thickening. Two esophagogastroduodenoscopies (EGD), one performed 1 year before admission and another performed 1 month before admission, showed non-specific mucosal changes. Also, 1 month before admission, a colonoscopy was performed to investigate diarrhea, which revealed no gross abnormalities; however, histology results of biopsies taken confirmed a diagnosis of collagenous colitis. Because of the known association between collagenous colitis and collagenous gastritis, his ongoing upper gastrointestinal symptoms prompted a third EGD, 9 days before admission, with biopsies. Superficial mucosal biopsies revealed active inflammatory mucosa with regenerative foveolar hyperplasia and scattered lamina propria eosinophils; however, a specific diagnosis was not determined. However, vonoprazan was discontinued because it was considered a potential cause of collagenous colitis. This discontinuation led to the resolution of diarrhea by the time of admission; however, nausea and vomiting persisted.

On admission, the patient was unable to tolerate oral intake and appeared malnourished and dehydrated. His body mass index was 18.6 kg/m^2^. Physical examination revealed sunken eyes, dry tongue and oral mucosa, reduced skin turgor, and bilateral pitting edema of the lower limbs. Cardiopulmonary, abdominal, and neurological examinations were unremarkable.

Laboratory test results indicated hypoalbuminemia (2.3 g/dL) and hypoproteinemia (total protein, 3.8 g/dL) with otherwise preserved liver function, coagulation, and total cholesterol (165 mg/dL). The blood glucose and glycated hemoglobin levels were 283 mg/dL and 11.7%, respectively. The hemoglobin level was elevated (18.0 g/dL), likely because of hemoconcentration. The platelet count was normal (349,000/μL). Urinalysis indicated only mild proteinuria (protein-to-creatinine ratio, 0.14 g/g Cr). A venous blood gas analysis excluded diabetic ketoacidosis.

All glucose-lowering agents, including GLP-1RA, were withheld to rule out drug-induced causes of nausea and vomiting. Although intensive insulin therapy was initiated after admission, gastrointestinal symptoms persisted; therefore, medication-related adverse effects were unlikely. We adopted a diagnostic approach focused on severe hypoproteinemia. Preserved hepatic synthetic function and imaging ruled out liver cirrhosis, and the absence of significant proteinuria excluded nephrotic syndrome. Malnutrition and protein-losing gastroenteropathy remained possible causes. Additional tests showed decreased immunoglobulins (Igs) (IgG, 218 mg/dL; IgA, 84 mg/dL; IgM, 11 mg/dL), ceruloplasmin, copper, zinc, and fat-soluble vitamins (25-hydroxyvitamin D, vitamin K_2_). This pattern of marked hypoalbuminemia with reduced Igs and multiple protein-bound factors suggested gastrointestinal loss rather than primary malnutrition, consistent with protein-losing gastroenteropathy. In the context of gastric wall thickening, Ménétrier’s disease was considered the leading diagnosis. Although collagenous colitis can rarely cause protein-losing gastroenteropathy, nausea and vomiting persisted despite diarrhea resolution.

**Figure 1:**
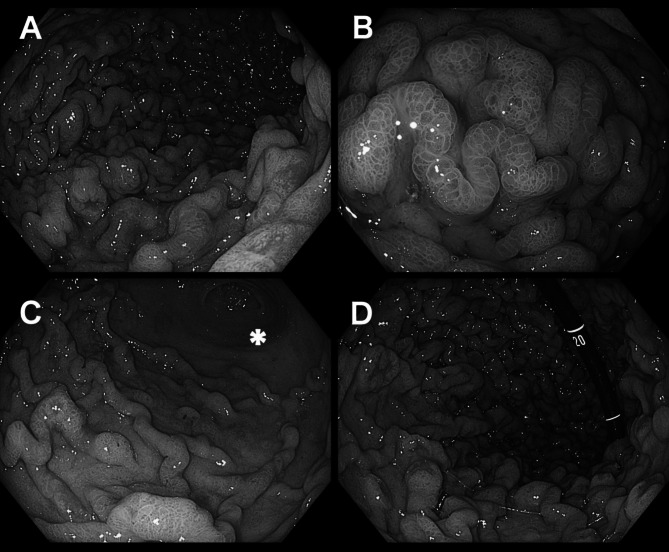
Esophagogastroduodenoscopy findings. **A**–**D** Images showing thickened gastric folds in the body and fundus with a cerebriform, edematous appearance and increased mucus production sparing the antrum (indicated by an asterisk).

**Figure 2:**
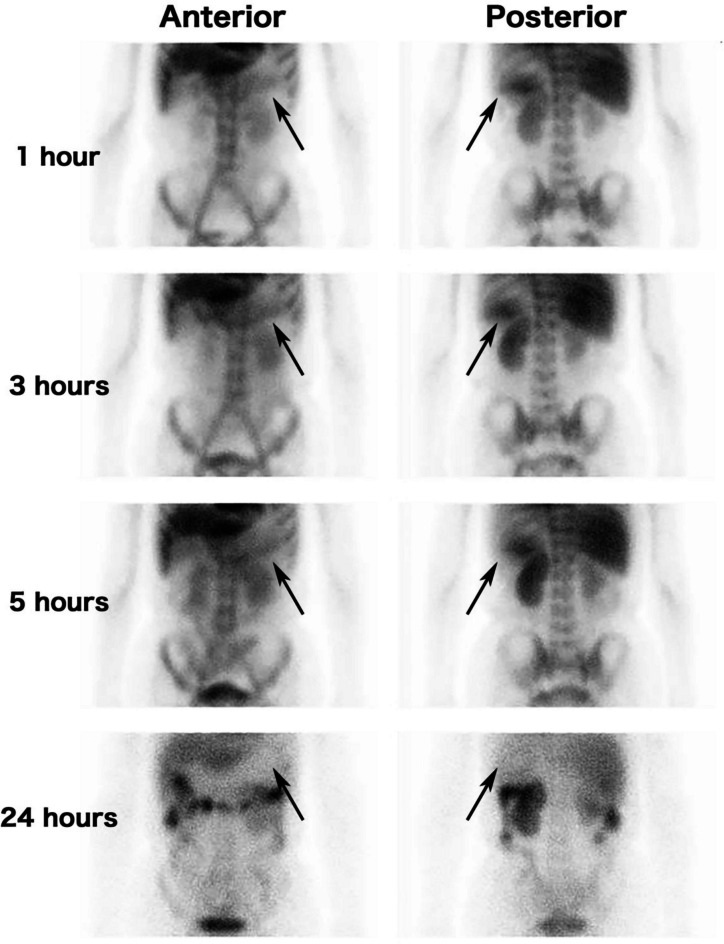
Serial ^99^mTc-human serum albumin scintigraphy images showing protein leakage into the upper gastrointestinal tract. Faint protein leakage into the upper gastrointestinal tract, particularly the stomach, was observed 1 h after administration (arrows). The leakage progressively increased. By 24 h, the protein was washed out from the upper tract. Accumulation was confined to the lower gastrointestinal tract, indicating significant protein loss primarily from the upper gastrointestinal tract.

**Figure 3:**
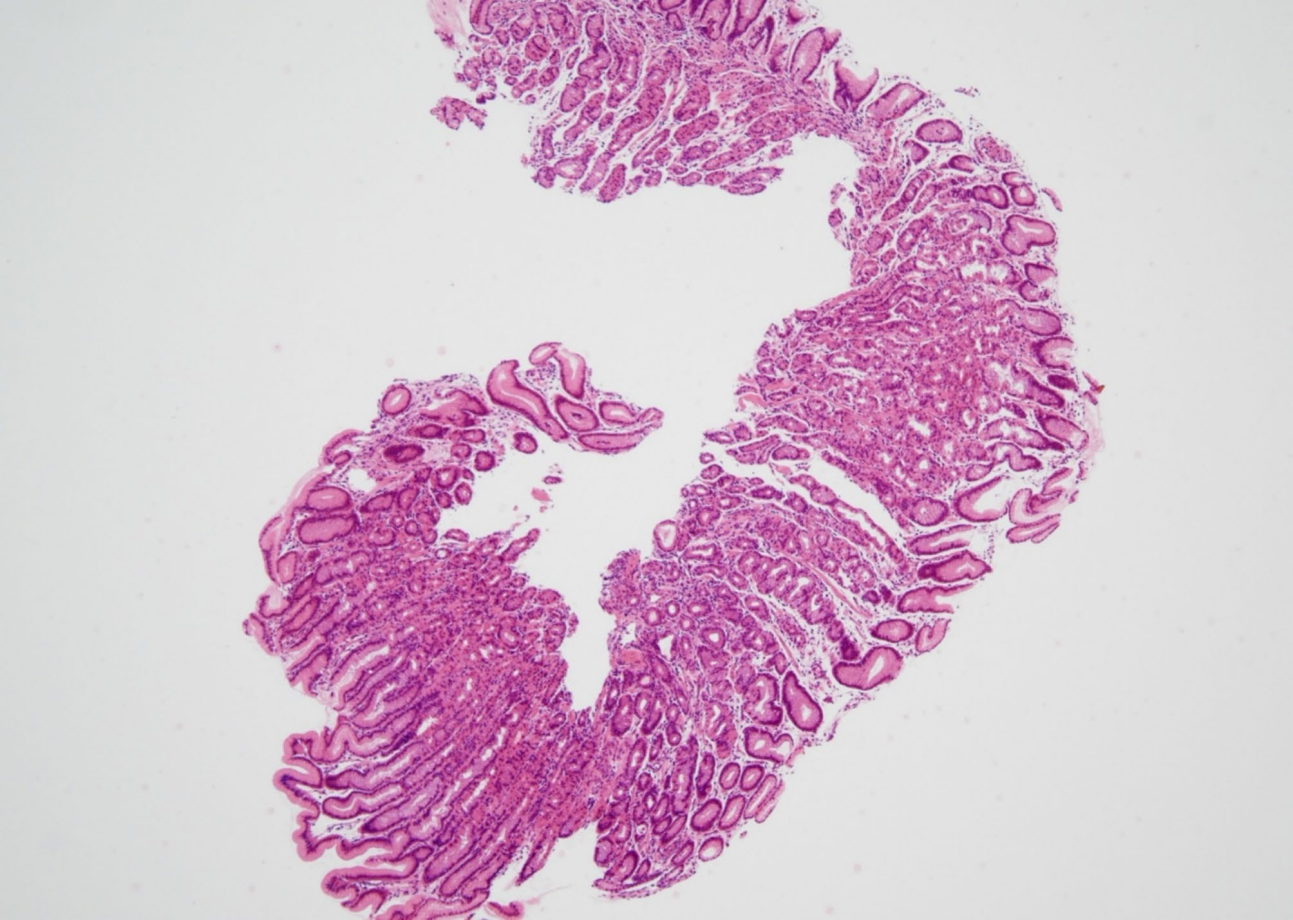
Histopathological findings of the gastric mucosa. Deep mucosal biopsy findings of the gastric body showing characteristic features of Ménétrier’s disease, including marked foveolar hyperplasia, cystic dilatation of glands, and diffuse glandular atrophy with loss of parietal and chief cells as well as interlacing smooth muscle bundles extending through the mucosa, which are characteristic of the deep mucosa, are shown. No evidence of malignancy is observed. Hematoxylin and eosin stain, original magnification ×100.

After admission, the first EGD on day 16 revealed giant cerebriform gastric folds in the gastric body sparing the antrum. A deep mucosal biopsy of the body was performed (Fig. 1). On day 21, ^99^mTc-human serum albumin scintigraphy revealed protein leakage confined to the upper gastrointestinal tract (Fig. 2). A mildly elevated serum gastrin level (77.8 pmol/L) suggested relative hypochlorhydria. *Helicobacter pylori* and cytomegalovirus (CMV) tests were negative. Histopathologic examination of the deep mucosal biopsy sample revealed marked foveolar hyperplasia, cystic dilatation of glands, diffuse oxyntic gland atrophy, and interlacing smooth muscle bundles extending through the full mucosal thickness without evidence of malignancy (Fig. 3). These findings were consistent with Ménétrier’s disease and effectively excluded differential diagnoses such as PPI-associated and P-CAB-associated gastropathy, infections (including *H. pylori* and CMV), hyperproliferative conditions (including Zollinger–Ellison syndrome and gastrointestinal stromal tumor), storage diseases (including amyloidosis), and malignancies.

**Figure 4:**
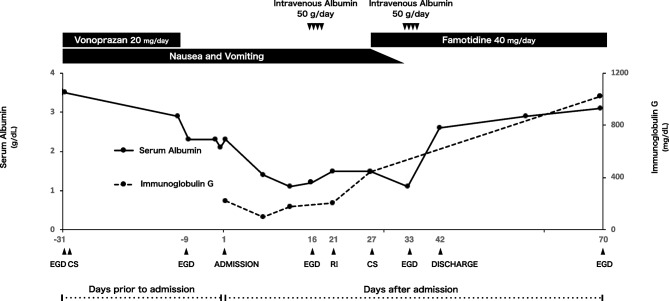
Clinical course showing serum albumin (g/dL) and immunoglobulin G (mg/dL) levels over time. Significant improvement in the levels of both markers is observed after famotidine treatment. Abbreviations: CS, colonoscopy; EGD, esophagogastroduodenoscopy; RI, radionuclide imaging (serial ^99^mTc-human serum albumin scintigraphy).

Nutritional support comprising small, frequent meals and high-protein foods enriched with medium-chain triglyceride oil (soup, biscuits) was initiated; however, postprandial vomiting persisted. Progressive severe hypoalbuminemia (nadir, 1.1 g/dL) led to worsening peripheral edema and orthostatic hypotension, which improved after intravenous albumin administration. On day 28, a repeat colonoscopy was performed. On day 31, histologic remission of collagenous colitis was confirmed, and famotidine (20 mg twice daily) and supportive acid suppression for Ménétrier’s disease were initiated. Within 4 to 5 days, nausea and vomiting resolved completely and he resumed normal oral intake. Follow-up EGD on day 35 revealed unchanged giant gastric folds; however, diffuse mucosal erythema was improved. Clinical improvement continued, and he was discharged on day 43. During the 1-month outpatient follow-up evaluation, serum albumin and IgG levels were nearly normalized, indicating remission of protein-losing gastroenteropathy (Fig. 4). Subsequent follow-up EGD demonstrated reduced gastric fold thickening.

## DISCUSSION

This is the first reported case of co-existing Ménétrier’s disease and collagenous colitis in a patient with refractory gastrointestinal symptoms and hypoproteinemia. It highlights diagnostic challenges posed by competing hypotheses.

Ménétrier’s disease is a rare hypertrophic gastropathy characterized by gastric mucosal hyperplasia and barrier dysfunction that leads to protein loss.^1,2^ No established criteria exist; therefore, the diagnosis relies on correlating clinical, endoscopic, and histopathologic findings after excluding mimickers.^3^ In this case, the onset of Ménétrier’s disease was considered insidious. Clinically apparent protein loss emerged only later during its course and contributed to a non-specific clinical presentation. Because Ménétrier’s disease is rare, early consideration was overlooked, and the patient was empirically treated with acid suppressants, mainly P-CABs, for presumed reflux disease or functional dyspepsia. After histologic confirmation of collagenous colitis 7 days before admission, his persistent upper gastrointestinal symptoms were subsequently attributed to an alternative diagnosis.^4,5^ Recognition of hypoalbuminemia and hypoproteinemia prompted diagnostic reconsideration.

Our clinical reasoning process focused on confirming protein-losing gastroenteropathy and identifying its source. The first EGD performed after admission revealed markedly enlarged gastric folds, which prompted further evaluation to determine a gastric cause of protein loss. Subsequent ^99^mTc-human serum albumin scintigraphy, performed during clinical remission of collagenous colitis, demonstrated protein leakage confined to the upper gastrointestinal tract, thereby excluding lower gastrointestinal sources and redirecting diagnostic attention to gastropathy. Histopathologic examination of deep gastric biopsy samples obtained during EGD confirmed foveolar hyperplasia, cystic dilatation, glandular atrophy, and interlacing smooth muscle bundles characteristic of Ménétrier’s disease. No infectious triggers such as *H. pylori* or CMV were identified. Deep mucosal biopsy is indispensable for the diagnosis of Ménétrier’s disease because characteristic changes are often confined to deeper layers. Superficial biopsies may reveal only non-specific alterations. For example, prolonged use of P-CABs can induce hypochlorhydria with superficial mucosal changes resembling those of Ménétrier’s disease; however, they can be distinguished histologically by deep mucosal findings. Moreover, the mildly elevated serum gastrin level in this case was not typical of drug-induced gastropathy. Drug-induced hypochlorhydria resulting from PPI or P-CAB use usually produces marked hypergastrinemia (often > 2000–3000 pg/mL; median, approximately 400–900 pg/mL); however, our patient’s gastrin level was mildly elevated.^6^

The multifactorial pathogenesis of collagenous colitis, a subtype of microscopic colitis characterized by chronic watery diarrhea and a thickened subepithelial collagen band,^7^ likely involves mucosal immune responses to luminal antigens, medication triggers (such as PPIs and P-CABs), and autoimmune factors.^8–11^ Because clinical remission and histological remission occurred in our patient following vonoprazan discontinuation, we suspected that collagenous colitis was induced by P-CAB use, as previously reported.^10,11^ Although protein-losing gastroenteropathy is uncommon in collagenous colitis, rare cases have been reported.^12^ Increased mucosal expression of vascular endothelial growth factor (VEGF) may contribute to albumin leakage and subepithelial collagen deposition by enhancing vascular permeability and fibrogenesis.^13^ In this case, however, ^99^mTc-human serum albumin scintigraphy after clinical remission of collagenous colitis demonstrated protein leakage confined to the upper gastrointestinal tract; therefore, collagenous colitis was an unlikely primary source of protein loss.

A notable aspect of this case was the apparent therapeutic response of Ménétrier’s disease to famotidine (an H2RA). Despite inconsistent evidence, H2RAs can improve the gastric epithelial barrier function.^14–18^ Ménétrier’s disease is thought to involve transforming growth factor-α/epidermal growth factor receptor (EGFR) signaling pathway activation, which induces selective proliferation of surface mucous cells in the gastric body and fundus.^19^ This pathogenic mechanism is supported by both experimental models and the clinical efficacy of EGFR-targeted therapies such as cetuximab (a monoclonal antibody).^19–21^ However, Ménétrier’s disease may be a heterogeneous disorder with different upstream factors that converge on EGFR activation.^2^ *H. pylori* and CMV are recognized triggers of Ménétrier’s disease; however, co-occurrence with inflammatory bowel diseases such as ulcerative colitis is possible, and therapeutic responses vary accordingly, suggesting that alternative mechanisms can lead to EGFR activation.^22–24^ Variants such as foveolar hyperplasia-dominant and hypertrophic lymphocytic gastritis subtypes have been described.^25^ The favorable response to the H2RA in this case may reflect a disease subset more amenable to such therapy.

In Ménétrier’s disease, protein loss mainly results from paracellular protein leakage caused by the disruption of epithelial tight junctions.^26^ VEGF overexpression has been observed in some patients with Ménétrier’s disease, particularly within lamina propria mononuclear cells, suggesting a role in microvascular hyperpermeability and protein leakage.^27^ Although the role of VEGF in Ménétrier’s disease is incompletely understood, it may be a therapeutic target. H2RAs may suppress angiogenesis by blocking histamine-induced cyclic adenosine monophosphate-dependent protein kinase A (cAMP–PKA) signaling and downregulating endothelial adhesion molecules, thereby reducing VEGF transcription and vascular permeability. Experimental models have shown that histamine upregulates VEGF via H2R-dependent cAMP–PKA signaling, and that H2RAs downregulate endothelial adhesion molecules and reduce vascular permeability,^28^ potentially mitigating VEGF-driven hyperpermeability in Ménétrier’s disease. Confirmation of such mechanisms may support reconsidering H2RAs as low-cost, minimally invasive therapeutic options for selected subtypes.

## CONCLUSION

In this case, diagnostic attention was directed toward common disorders such as gastroesophageal reflux disease and functional dyspepsia, followed by collagenous colitis. Renewed attention to evolving gastric changes and hypoproteinemia broadened the clinical perspective and led to the diagnosis of Ménétrier’s disease. Persistent upper gastrointestinal symptoms accompanied by gastric wall thickening and hypoproteinemia should prompt consideration of this diagnosis. Although EGFR inhibitors are promising targeted therapies, H2RAs may play a therapeutic role in selected cases. Knowledge of the pathophysiology of acid secretion and endothelial growth factor activity can inform therapeutic options that have not yet been tested in clinical trials.

## Data Availability

Data sharing is not applicable to this article as no datasets were generated or analyzed during the current study.
